# Preliminary Results of Bone Lengthening over Telescopic Titanium Intramedullary Rod

**DOI:** 10.1155/2023/4796006

**Published:** 2023-01-30

**Authors:** Eduard Mingazov, Nikita Gvozdev, Arnold Popkov, Pierre Journeau, Dmitry Popkov

**Affiliations:** ^1^Russian Ilizarov Scientific Centre for Restorative Traumatology and Orthopaedics, Kurgan, Russia; ^2^Centre Hospitalier Universitaire de Nancy, Nancy, France; ^3^Faculty of Medicine, University of Kragujevac, Kragujevac, Serbia

## Abstract

**Background:**

Limb lengthening and deformity correction in patients with abnormal bone associating fragility often require an approach combining methods of external and internal fixation. This study demonstrates results of simultaneous application of external fixator, and telescopic rod for femoral lengthening and deformity correction in three children with osteogenesis imperfecta or severe form of Ollier's disease.

**Materials and Methods:**

Three patients (two boys with Ollier's disease and a girl with osteogenesis imperfecta, type I) were operated on for femoral lengthening with combined technique associating Ilizarov frame and titanium telescopic intramedullary rodding.

**Results:**

Planned amount of lengthening and deformity correction were achieved for all patients. We found neither rod bending nor pull out of threaded tips. There was no difficulty of expanding of telescopic intramedullary rods made of titanium alloy during distraction phase of lengthening procedure.

**Conclusion:**

This short series proved feasibility of performing one-stage surgery with external frame and telescopic rodding in limb lengthening. The technique of telescopic rods in lengthening procedure is promising method requiring meticulous insertion of rod in centralized positioning in epiphysis. Acute alignment of the segment been elongating should be achieved at surgery. No any progressive angular deformity correction in postoperative period is authorized in order to avoid bending of telescopic rod. This combined approach does not affect bone healing.

## 1. Introduction

In treatment of conditions associating bone fragility (e.g., osteogenesis imperfecta, Ollier's disease, polyostotic fibrous dysplasia, and metabolic disorders), surgeons often face secondary deformity and bowing, limb length discrepancy representing indications for bone lengthening, and alignment procedures where combined approaches demonstrate advantages [1–5]. An internal device left in situ for a long time or even a life is a crucial element because it reduces risks of deformity recurrence or pathologic fractures [6, 7]. Last years, some advanced technologies emerged, for example, fixator-assisted intramedullary nailing after lengthening [2, 8–10]. In patients with congenital limb length discrepancy and Ollier's disease, lengthening procedures over nail or fully implantable lengthening rods have been described as techniques, providing protection against refracture and enabling earlier rehabilitation [11–13]. However, growth zones in a segment to be lengthened in children limit use of conventional or electromagnetic rigid intramedullary rods [14]. Furthermore, these devices are not applicable in children or for lengthening of bones with small shaft diameter [12, 15, 16]. Furthermore, the electro-magnetic rods left in situ arise concerns about their long-term effect [17].

Schiedel et al. [8] and Grill et al. [9] proposed an approach of prophylactic stabilization of lengthened femur in children with regular rod or flexible intramedullary nailing (FIN)—“lengthening then rodding” [8, 9]. However, the risks of fracture at the moment of frame removal and insertion of a nail during additional surgery are not negligible [8]. Another disadvantage of the reported technique is the risk of infection due to the one-stage change from the external to the internal fixation [9]. Our findings and results of series patients with abnormal bone demonstrated that the FIN applied during initial surgery simultaneously with external frame allows to reduce healing index and complications related to external fixation, but being inserted through metaphyseal zones does not protect newly formed bone related to spontaneous growth [18, 19].

In pediatric orthopedics, a telescopic rod left in situ after deformity correction should be considered as the most important element of osteosynthesis in abnormal bone: its internal and external parts follows natural growth of a segment due to telescoping effect [3, 6, 20–22]. Popkov et al. [23] found that telescopic rodding has advantages over FIN in terms of survival rate and reduced number of reoperation [23]. Planning this study we speculated that being attached to proximal and distal epiphyses telescopic implant is likely to be retained for a much longer period following external fixator removal after bone lengthening in patients with abnormal fragile bone. This study demonstrates results of simultaneous application of external fixator and telescopic rod for femoral lengthening and deformity correction in three children with osteogenesis imperfecta or Ollier's disease.

## 2. Materials and Methods

Our study is a retrospective series of three femoral lengthening between March 2021 and May 2022 (Table 1). The mean age was 5.3 ± 0.82 years. Two patients with Ollier's disease were boys. Only one patient with dyschondroplasia was previously treated for tibial lengthening with Ilizarov system 1.5 years before admission.

All patients and their families complained about decreased walking ability or loss of this function, deformity of lower limb, and lower limb length discrepancy. Since birth, each patient faced 2–4 pathological fractures managed conservatively.

**Figure 1:**
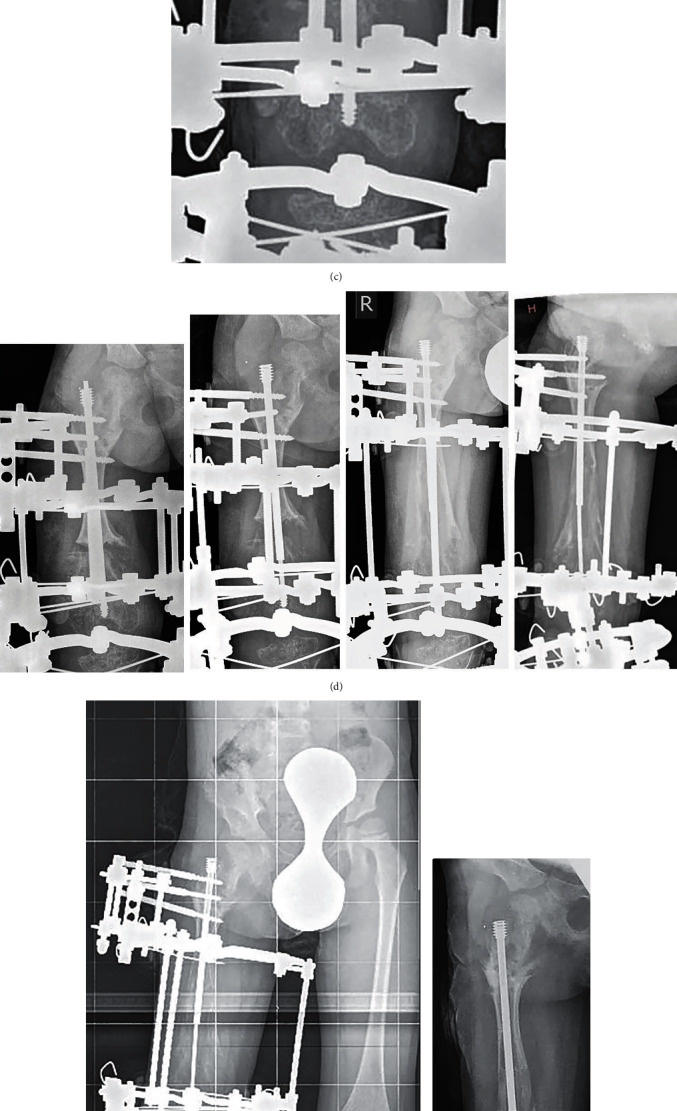
Radiographs of patient L.: (a) full-length standing radiograph and lateral view before surgery; (b) intraoperative radiograph performed after corrective osteotomy and insertion of the rod; (c) intraoperative radiograph done after frame application, by the end of surgery; (d) consecutive Antero-posterior (AP) radiographs demonstrating progressive lengthening of femur and bone union by the end of treatment period. Note excellent position of threaded ends of rods parts into distal epiphysis and apophysis of greater trochanter; (e) full-size standing radiograph of lower limb, satisfactory alignment of right limb; (f) position of the rod after frame removal: no rod migration neither bending. Threaded ends are into apophysis and distal epiphysis; (g) in three months after frame removal.

**Figure 2:**
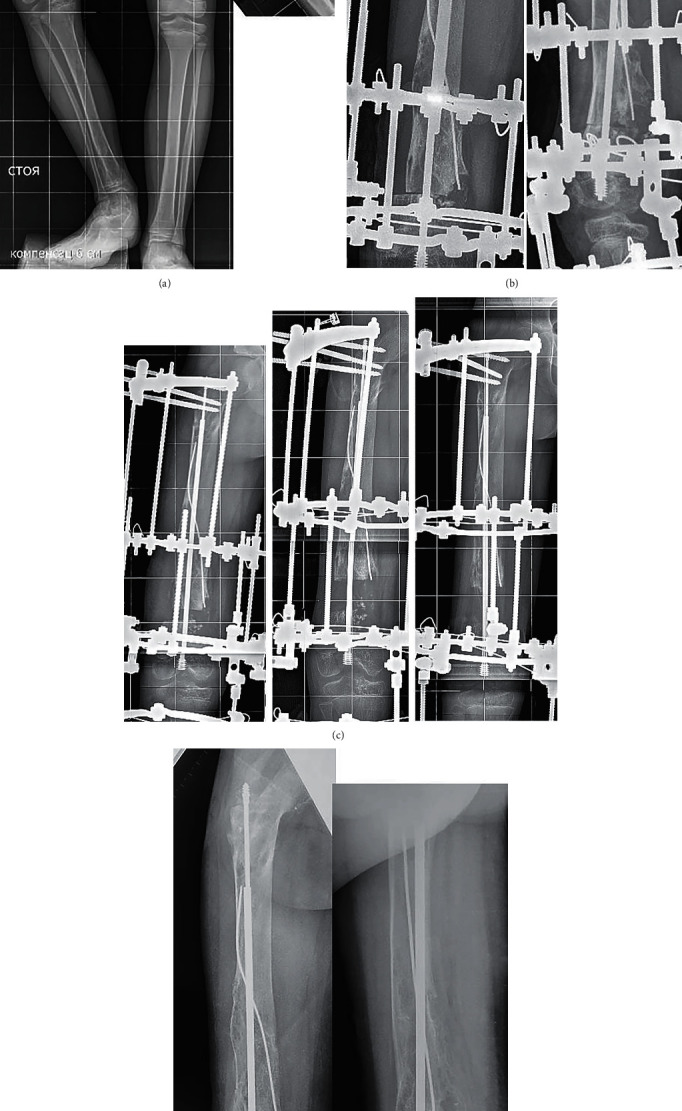
Retrograde telescoping rod for femoral lengthening in patent R.: (a) preoperative full-size standing radiograph and lateral view of the right femur. Note a voluminous distal cartilaginous zone; (b) intraoperative radiograph demonstrating retrograde position of rod and exact centration of knee joint center and mechanical femoral shaft axis; (c) consecutive radiographs of distraction period; and (d) after frame removal.

Surgical technique for lengthening over telescopic rod for children with enchondromatosis in our study consisted of several stages. In patients with Ollier's disease (Figures 1 and 2) surgical steps included removal of previous material (in one case) that was followed by osteotomy performed in an open technique. The site for osteotomy and amount of correction were determined preoperatively. We inserted a guidewire through osteotomy site under fluoroscopic guidance for reaming of femur in each case of Ollier's disease. After drilling only throughout intraosseous cartilaginous tissues aiming guide were removed. Then a guide wire was inserted through greater trochanter in one patient or through center of distal epiphysis (para patellar approach was used) in another patient. Once the bone was aligned and reamed, the guide wire was replaced by male rod. At this stage, the male rod was inserted only until opposite epiphysis/greater trochanter, but not screwed. The female rod was cut and then inserted over the male rod. Female rod was always screwed first in epiphysis/apophysis of femur. Then, the male rod was fixed into distal epiphysis in one patient (for antegrade insertion) or into greater trochanter in other patient (for retrograde insertion) under X-ray control (C-arm) using T-handle. In all patients, the titanium alloy telescopic rod (Intramedullary Telescopic Rod, reg. certificate no. RZN 2017/6876, Designed Metis Ltd., Tomsk, Russia) was used. The diameter of female rod was 5.5 mm in both cases. The antegrade or retrograde direction of insertion was done such a way that female part of a rod was placed into more voluminous enchondromatosis zone. At the final step of surgery, Ilizarov proximal short arc with 3 half-pins, middle ring with wire(s), and a half-wire and distal ring with 3–4 wires were applied in children with Ollier's disease. Rings and arc were connected. Rods of connection were always parallel to telescopic rod.

**Figure 3:**
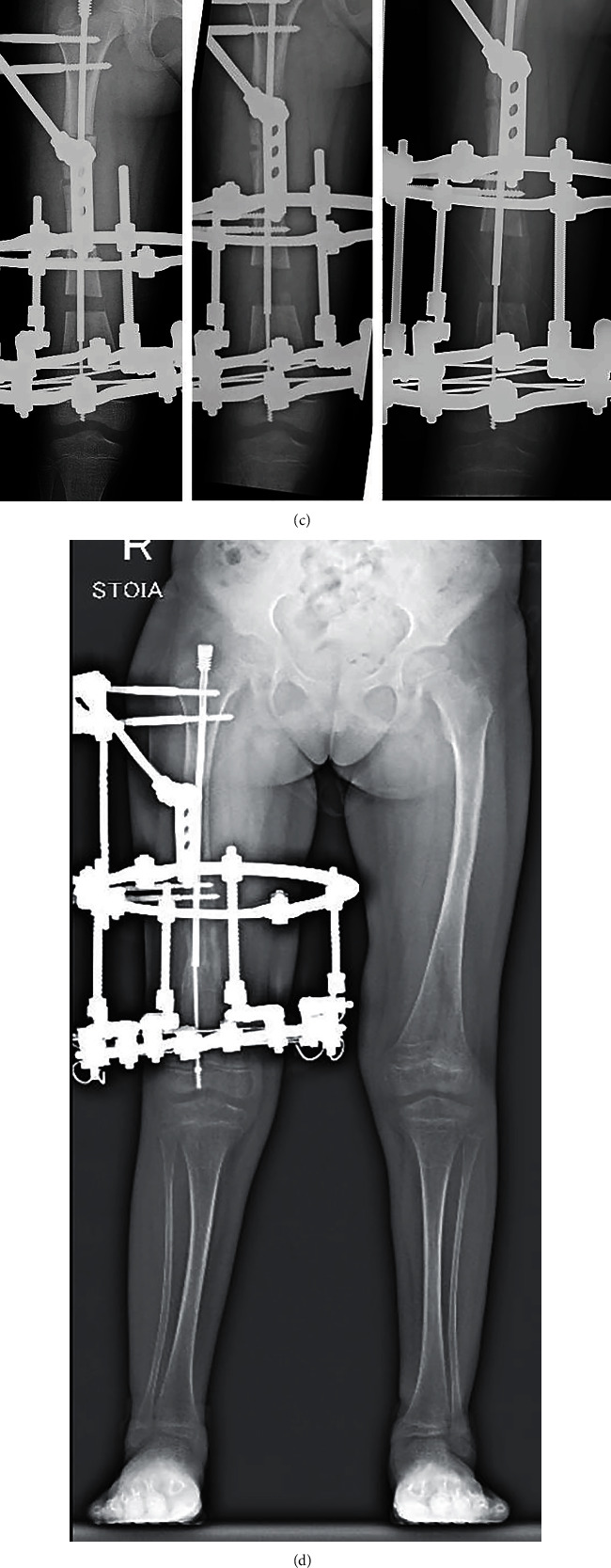
Patient S., osteogenesis imperfecta, type I, radiographs: (a) preoperative full-size standing radiographs; (b) intraoperative radiographs: double osteotomy of the right femur. Note open-wedge proximal osteotomy; (c) consecutive radiographs demonstrating increasing gap between fragments and developing bone regenerate during distraction period; (d) full-size standing radiograph of lower limbs, satisfactory alignment and lengthening of right limb; and (e) full-size standing radiograph of lower limbs in 3 months after frame removal, satisfactory alignment of the lengthened of right limb (anatomical position of the mechanical lower limb axis).

There were some difference in the surgical technique for girl with osteogenesis imperfecta (Figure 3). Percutaneous osteotomies were always performed under fluoroscopic guidance. For insertion and screwing of male and female parts, we followed suggestions of Birke et al. [3]. At proximal osteotomy site reduction of the varus deformity was automatically obtained while female part inserted. The diameter of titanium female rod was 4.2 mm. An Ilizarov frame was done as final step of surgery. Derotation at proximal osteotomy site was done acutely between proximal arc and middle ring. We consider it a strict rule that connecting rod for lengthening must be parallel to telescopic rod.

Patients started standing and walking with weight-bearing since second or third postoperative day. Distraction phase was initiated on fifth day with rhythm of 1.5 mm per day for the first 6–7 days followed by X-ray control. This slightly accelerated every-day distraction during initial phase ensured telescoping of rod parts avoiding risks of premature bone union. Once an interfragmentary gap evident, an every-day distraction was reduced up to 1 mm. During elongation period the distraction rate varied depending on bone regenerate intensity. Evident radiological signs of bone union justified frame removal.

## 3. Results

Amount of lengthening, healing index, time for external frame wearing, problems, and complications are represented in Table 2.

Planned lengthening was achieved in all patients. Orthosis or plaster cast with free hip and ankle joints was applied for 3–4 weeks after frame removal. Patients continued walking with progressive weight-bearing with the orthosis on.

Neither loss of threaded fixation in the distal femoral epiphyses and apophysis of the greater trochanter nor migration of the rod into the knee and ankle joints were observed in the patients. We found no blocking of telescoping rod during elongation phase neither secondary rotational bone displacement. Furthermore, distraction phase confirmed that male and female parts were not blocked by wires or half-pins of external fixator. No complications requiring unscheduled surgery were noticed. We observed one superficial pin-site infection managed with local therapy. No one wire or half-pin were removed before definitive frame removal.

## 4. Discussion

In pediatric orthopedics, an intramedullary device (including telescopic one) left in situ after deformity correction and/or lengthening in conditions of abnormal bone is an important element to maintain the bone straight during growth, reduce fracture rate, and facilitate fracture management in long-term follow-up [2–7].

The approach “lengthening then rodding” represents disadvantages of a manipulation of the bone during frame removal or implantation of the regular rods [8] and the risk of infection [9]. A multicenter study emphasizes advantages of an approach associating intramedullary device and external fixation at the primary surgery for bone lengthening in children with abnormal bone diseases [24], [25].

We may reveal from multiple recent publications that in patients with osteogenesis imperfecta the telescopic rodding ensures fewer complications, reduced need for surgical revision, and more optimal outcomes in comparison to regular rodding/nailing or even transphyseal sliding FIN [20, 23, 24].

We recognize that it is difficult to draw a large conclusion from our small heterogeneous group of patients operated on with different approach and short-term follow-up. That is why the focus of our discussion is on surgical aspects, and potential pitfalls when using telescoping rod for abnormal bone lengthening with an external device.

A telescopic rod can be prone to rod part migration, limited telescoping, and rod bending in patients with osteogenesis imperfecta [3, 5, 26–28]. Holmes et al. emphasize appropriate positioning of distal threaded part in femoral epiphysis as crucial element of technique aiming to increase rod survival: central positioning of the rod in the distal and fixating the distal tip increase the longevity of the rod [27]. A bending positioning represents a mechanism of rod pull out leading to rod failure complications [27, 29–31]. An additional aligning osteotomy keeps its recommendations to facilitate rodding and avoid bending of rod [7, 31].

At the stage of planning of our new approach, we anticipated those problems and complications potentially amplified by increased speed of rod telescoping due to conventional bone lengthening distraction rate and direction of bone fragment elongation.

In new lengthening conditions of distraction phase a minimal initial bending of rod amplifies friction force and could result in limited or failure telescoping leading to disengagement of threaded parts. In our series, the connecting rods of external frame parallel to telescopic rod allowed to avoid three-point forces bending, and thereby to prevent telescoping failure. We could draw a suggestion that the perfect alignment should be obtained during surgery, and direction of further elongation should be parallel to axis of the intramedullary rod. No any progressive angular deformity correction in postoperative period can be authorized if this surgical technique applied. The following of these rules allowed in our series to avoid telescoping failure and loss of fixation of threaded tips even during distraction phase. We would like to emphasize that no difficulties of expanding of telescopic intramedullary rod made of titanium alloy were found. Thereby, this short series proves feasibility of performing one-stage surgery with external frame and telescopic rodding in limb lengthening aimed to protect a lengthened segment after frame removal by telescopic rod left in situ. This approach allows to avoid risk of two-staged method called “lengthening then rodding.”

To avoid the pitfall of intraoperative joint-intrusion we followed the suggestions of Birke et al. [3] and never screwed male component first. There were no knee joint intrusions by male rod in our series. All threads stayed intraosseous during distraction phase and after frame removal.

The only use of telescoping rod in abnormal bone does not sufficiently stabilizes the positioning of bone fragments and can be complicated with secondary rotational and/or longitudinal displacement [3, 4, 32, 33]. Cho et al. [4] and Franzone and Kruse [34] use a short locked plate as additional element of fixation permitting to overcome those risks [4, 34]. An external frame can also be applied for this purpose [5]. In actual series, Ilizarov frame was applied not only to avoid the secondary displacement but to enable abnormal bone lengthening. Despite an intramedullary device, we found no problems for bone healing. Our healing index is comparable to those ones reported in previous publications varying from 19.5 to 28.8 days/cm [19, 35–37].

There were no significant complications like deep infection, nerve injury, and non-union caused by external fixation.

One limitation of our study is a small group. Another limitation is the heterogeneity of our cohort: patients varied in age and type of pathology conditioning abnormal bone. Because of short follow-up we do not report rate of rod failure in middle, and long-term follow-up where rod bending can be caused by the growth of the patient.

## 5. Conclusion

This short series proved feasibility of performing one-stage surgery with external frame and telescopic rodding in limb lengthening. The both techniques for telescopic rodding and external fixation, and their association are demanding and the experience of the surgeon is not negligible. The use of telescopic rods for lengthening procedure is promising method requiring meticulous insertion of rod in centralized positioning. Acute alignment of the segment been elongating must be achieved at surgery. No any progressive angular deformity correction in postoperative period is authorized in order to avoid bending of telescopic rod. An “accelerated” expanding of titanium telescopic rod during lengthening procedure did not result in pull out of threaded tips and telescoping failure in our series. This combined approach did not affect bone healing.

## Figures and Tables

**Table 1:** Patients demographic data and type of surgery.

Patient	Diagnosis	Age	Type of surgery with rod	Telescopic rod	Simultaneous surgery
S.	Osteogenesis imperfecta, type I	5 years and 2 months	Bifocal osteotomy of right femur (proximal shaft site—Acute torsion and open wedge varus deformity correction, distal shaft site—Progressive lengthening)	Titanium telescopic rod of 4.2 mm diameter, antegrade insertion in right femur	Ilizarov frame
L.	Ollier's disease	4 years and 9 months	Distal femoral shaft osteotomy, acute torsional and varus (closed wedge) deformity correction	Titanium telescopic rod of 5.5 mm diameter, antegrade insertion in right femur	Ilizarov frame simultaneous tibial lengthening over elastic nails
R.	Ollier's disease	7 years and 4 months	Distal femoral metaphysis closed wedge osteotomy, acute varus deformity correction	Titanium telescopic rod of 5.5 mm diameter, retrograde insertion in right femur	Ilizarov frame, removal of one elastic nail from femur

**Table 2:** Results of lengthening over telescoping rod.

Patient	Amount of lengthening (cm)	Amount of lengthening (%)	External frame wearing (days)	Healing index (days/cm)	Complications	Residual superposition of rod parts (%)
S.	4.2	16.7	121	28.8	None	61.8
L.	5.8	35.7	113	19.5	Superficial pin site infection (local treatment)	43.9
R.	4.0	15.4	97	24.3	None	69.9

## Data Availability

Data supporting this research article are available from the corresponding author on reasonable request.
